# Congenital and Iatrogenic Esophageal Diverticula in Infants and Children: A Case Series of Four Patients

**DOI:** 10.7759/cureus.68806

**Published:** 2024-09-06

**Authors:** Delvis J Garcia, Abdulqadir J Nashwan, Amani N Al-Ansari

**Affiliations:** 1 Department of Pediatric Surgery, Hamad Medical Corporation, Doha, QAT; 2 Department of Pediatric Surgery, Jose Marti y Perez Pediatric Teaching Hospital, Sancti Spiritus, CUB; 3 Nursing & Midwifery Research Department, Hamad Medical Corporation, Doha, QAT; 4 Department of Surgery, Hamad Medical Corporation, Doha, QAT

**Keywords:** atresia, diverticulum, esophagogram, esophagus, pediatric, zenker’s diverticulum

## Abstract

In pediatric patients, esophageal diverticulum (ED) is rare and can be severe, especially when involving the cervical esophagus. Diagnosis and treatment typically start after birth, with some cases managed conservatively. This series presents four ED cases from Jose Marti y Perez Pediatric Teaching Hospital in Cuba (2003-2020). Symptoms included difficulty swallowing, regurgitation, and breathing problems. Three cases required surgery: a five-month-old post-esophageal-coloplasty (managed conservatively), a four-year-old post-esophageal atresia repair (diverticulum partially used to fix a narrow spot), and a 16-year-old with Zenker's diverticulum (requiring surgical removal). A 35-day-old baby with ED post-type C esophageal atresia surgery died from a pre-existing condition. Surviving patients lived healthy lives. ED in pediatrics can be congenital or iatrogenic post-esophageal repair. Reflux symptoms, respiratory distress, or a cervical mass should prompt suspicion of ED.

## Introduction

Esophageal diverticulum (ED) in pediatrics has significant implications, highlighting the importance of early detection and management [1]. ED presents with respiratory distress, poor feeding, and inflammation, affecting nutrition status and general health [2]. While ED is a concern among the elderly and middle-aged, this esophageal anomaly is rare in newborns, infants, and children [3]. Further, this medical condition and its potential therapies are seldom addressed in pediatric medical literature [4].

In children, ED may be congenital or iatrogenic as a consequence of surgery on the esophagus [5]. Congenital ED is exceptionally rare in neonates and premature babies and occasionally presents alongside other esophageal anomalies, such as esophageal atresia or trachea-esophageal fistula [6]. Zenker’s diverticulum (ZD) in newborns is typically characterized by stridor and poor feeding [7].

In the pediatric population, true ED, involving all esophageal wall layers, is seldom encountered in clinical practice [8]. However, surgical literature reports cases of ED that occur postoperatively as complications following esophageal atresia and tracheoesophageal fistula surgery [9]. Estimates suggest postoperative ED in cases of up to 1.9% [10-11]. Post-esophageal surgical ED is usually distinct from the primary type. The etiology of primary ED is a pulsion type due to esophageal dysmotility, obstruction, or esophageal wall weakness [12]. Conversely, secondary ED is mainly a traction type due to peri-esophageal inflammation [13]. This case series reports these two types of ED in pediatrics, emphasizing the diagnosis and management of the associated complications.

## Case presentation

The cases presented originate from Jose Marti y Perez Pediatric Teaching Hospital in Sancti Spiritus province, Cuba, between 2005 and 2020. Consent for ethical clinical procedures and verbal consent for publication were obtained. Patient data were meticulously collected from the hospital’s medical records archive. Precaution has been taken to ensure patient identifiers remain confidential throughout this case series reporting.

Case 1 (2003)

A five-month-old female child presented to the emergency department with accidental ingestion of an alkaline substance used for domestic purposes. After resuscitation, an esophagoscopy was performed 26 hours after admission. The endoscopic examination revealed grade 3B esophagitis, as classified by Zargar’s classification. The most affected areas were in the esophagus's middle and lower third.

Three days later, the patient developed lower esophageal perforation, leading to mediastinitis and right hydropneumothorax. Timely cervical esophagostomy, gastrostomy, and right pleural cavity drainage were performed. The patient exhibited a favorable clinical course with no significant adverse events. After 45 days of admission, the patient was discharged. Due to the child's recurrent, unresolved bronchopneumonia and challenges in achieving adequate nutritional status, the child was placed on gastrostomy feeding until the age of two, as reported by the family.

When the patient's respiratory, general, and nutritional state improved at this age, a retrosternal esophagocoloplasty was conducted. The left colon in an isoperistaltic position was used as a replacement for the esophagus. The surgeons utilized the left colic artery vascular pedicle as the blood supply. Postoperatively, mild stenosis at the esophageal-colic anastomosis occurred and was responsive to dilatation.

Three years postoperative, the patient suffered from intermittent dysphagia, noisy swallowing, and regurgitation of undigested food. Esophagography showed a 3-cm rounded bag arising from the posterior aspect of the pharyngoesophageal part of the remaining esophageal segment. The condition was diagnosed as type 3 Zenker’s diverticulum as classified by Brombart’s classification. Surgical intervention was planned; however, the family preferred conservative treatment.

**Figure 1:**
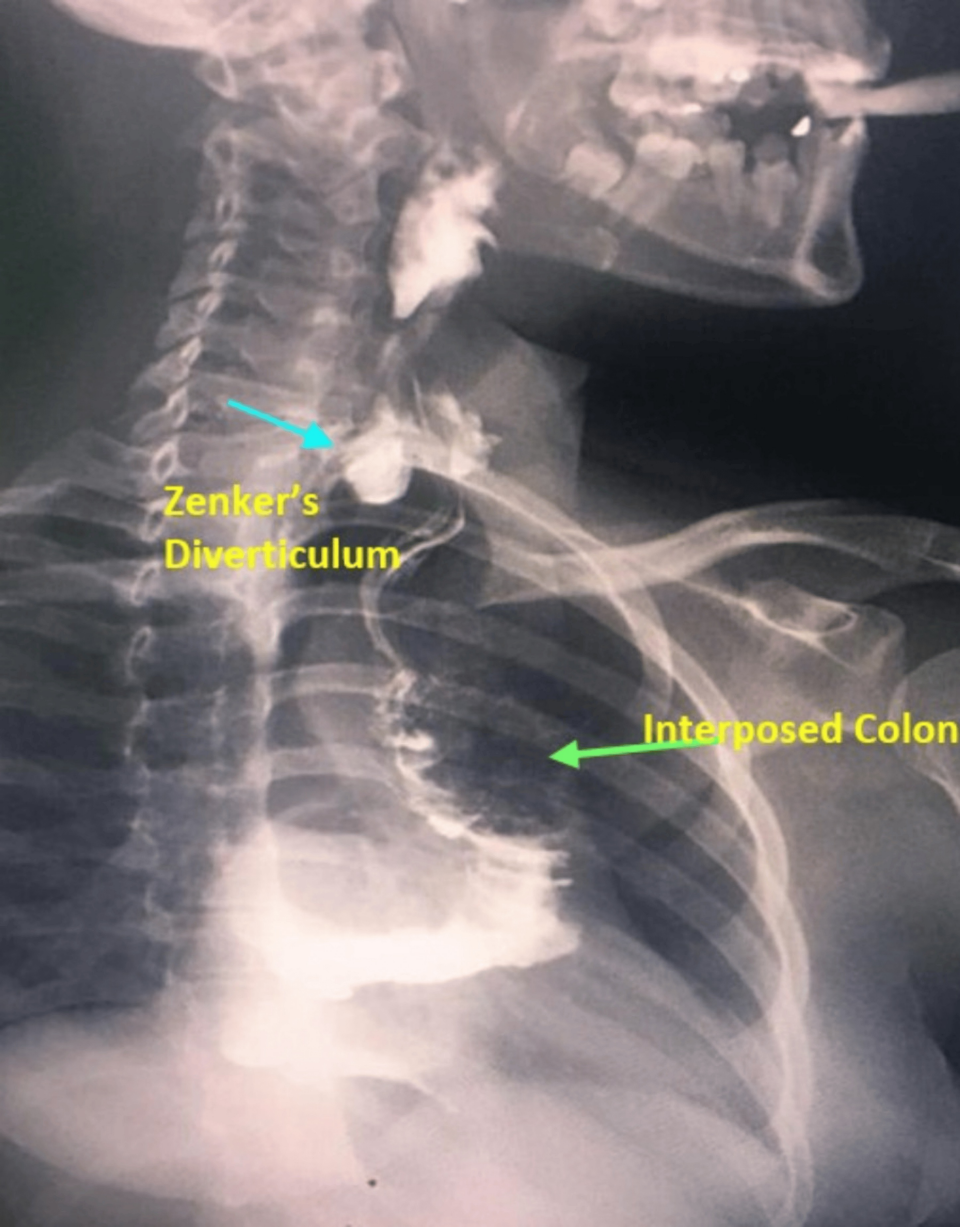
Case 1: Esophagogram showing Zenker’s diverticulum (blue arrow) and the interposed colon (green arrow).

**Figure 2:**
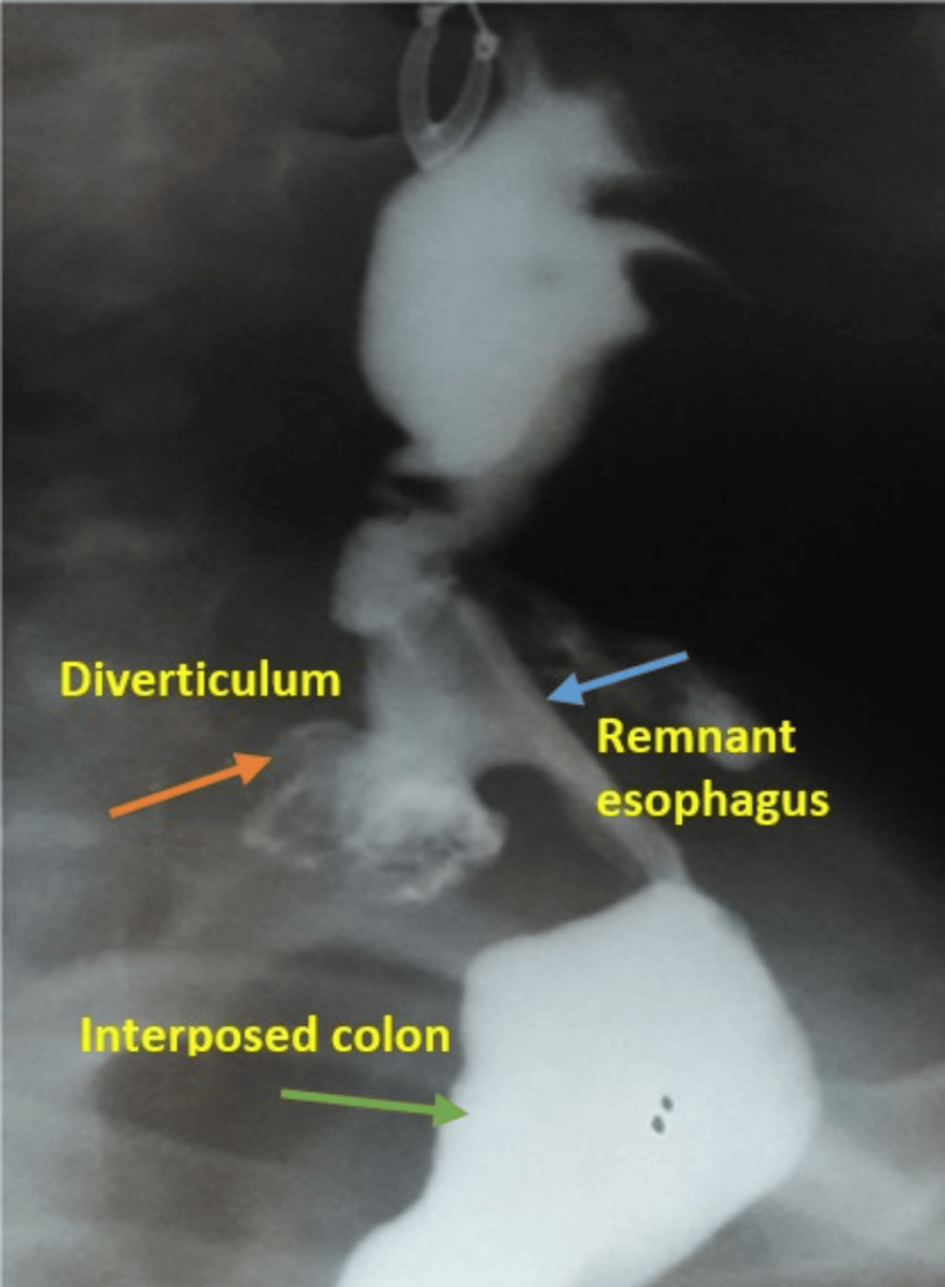
Case 1: Barium swallow showing leakage (arrow) of the contrast inside the diverticulum lumen.

Given the wide diverticular stoma, the patient could perform self-managed maneuvers to empty the diverticulum. The patient’s diet was maintained as a hard diet. The patient had regular follow-ups until the age of 20. During the last follow-up, we observed that the patient was doing well with moderate symptoms. Radiological evaluation showed no increase in the diverticulum size (Figures 1, 2).

Case 2 (2005)

A four-year-old female child presented with intermittent dysphagia and reflux symptoms, including heartburn and regurgitation. The parents reported a history of esophageal atresia and tracheoesophageal fistula since birth. The fistula was ligated, and the esophagus was repaired by end-to-end primary esophageal anastomosis. The patient was doing well, receiving only treatment for esophageal reflux. A diverticular sac protruding from the esophageal wall, proximal to anastomosis, was observed upon radiological evaluation. A diagnosis of mid-esophageal diverticulum was confirmed.

During surgery, the anatomical position of the diverticulum was radiologically confirmed. Additionally, the diverticulum was adherent to the posterior tracheal wall at the site of the origin of the right main bronchus by a short fibrous path. This fibrous attachment was presumed to be related to the previous primary tracheoesophageal fistula. Consequently, the fibrous path was resected. The integrity of the tracheobronchial wall was ensured. No patency was found between the esophageal and tracheobronchial lumen. The diverticulum was partially excised. The distal portion was then used as a flap, to widen the esophageal lumen to correct the moderate stenosis at the suture site. Pathological examination of the excised specimen showed a complete esophageal wall with all its layers (true diverticulum). Postoperatively, the patient had no more dysphagia and was maintained on anti-reflux medications. The five-year follow-up was uneventful.

Case 3 (2007)

A 16-year-old female patient was admitted to the hospital with severe inflammation in the left cervical region persistent for two weeks. The patient exhibited poor general condition in addition to symptoms such as dysphagia, dysphonia, high fever, and dehydration. She had a history of heartburn, episodes of dysphagia, and regurgitation for more than three years without receiving proper medical attention.

Upon conducting a general examination, a septic condition was suggested. Local examination revealed an almost 5-cm swelling in the left cervical region anterior to the sternocleidomastoid muscle. The swelling appeared erythematous, hot, and tender, with a fibrin crust covering a central area showing necrotic changes. Upon removal of the crust, fetid purulent thick content emerged, followed by copious secretion, which strongly suggested the diagnosis of an infected diverticulum.

General measures were implemented to address the septic condition, including fluid administration and empiric broad-spectrum antibiotics to cover the typical local flora. The swelling was drained using Penrose drainage, and periodic dilatation of the cervical fistula orifice was performed.

After the patient’s condition stabilized, esophagography was conducted 48 hours after admission. Radiological examination revealed a bag-shaped contrast accumulation in the left prevertebral space. The contrast was observed leaking through the cervical drainage orifice with minimal passage to the distal esophagus, confirming a diagnosis of Zenker’s diverticulum. The poor passage of contrast to the distal esophagus was attributed to extrinsic compression exerted by the inflamed diverticulum, causing the severe symptoms presented by the patient. The abscess drainage and antibiotic treatment restored the patient's ability to swallow.

After undergoing surgical drainage and receiving medical treatment, the patient recovered satisfactorily. After 10 days, the patient was stepped down from the ICU to the general ward room and discharged home after 30 days. A diverticulectomy and cricopharyngeal muscle ablation were performed with improvement in the patient’s general condition and nutritional status through a left cervical approach. Five days postoperative, a salivary low-flow fistula appeared at the drainage site and healed spontaneously within one week with no further complications. The patient was discharged on the 17th postoperative day and 20 days after hospital admission.

**Figure 3:**
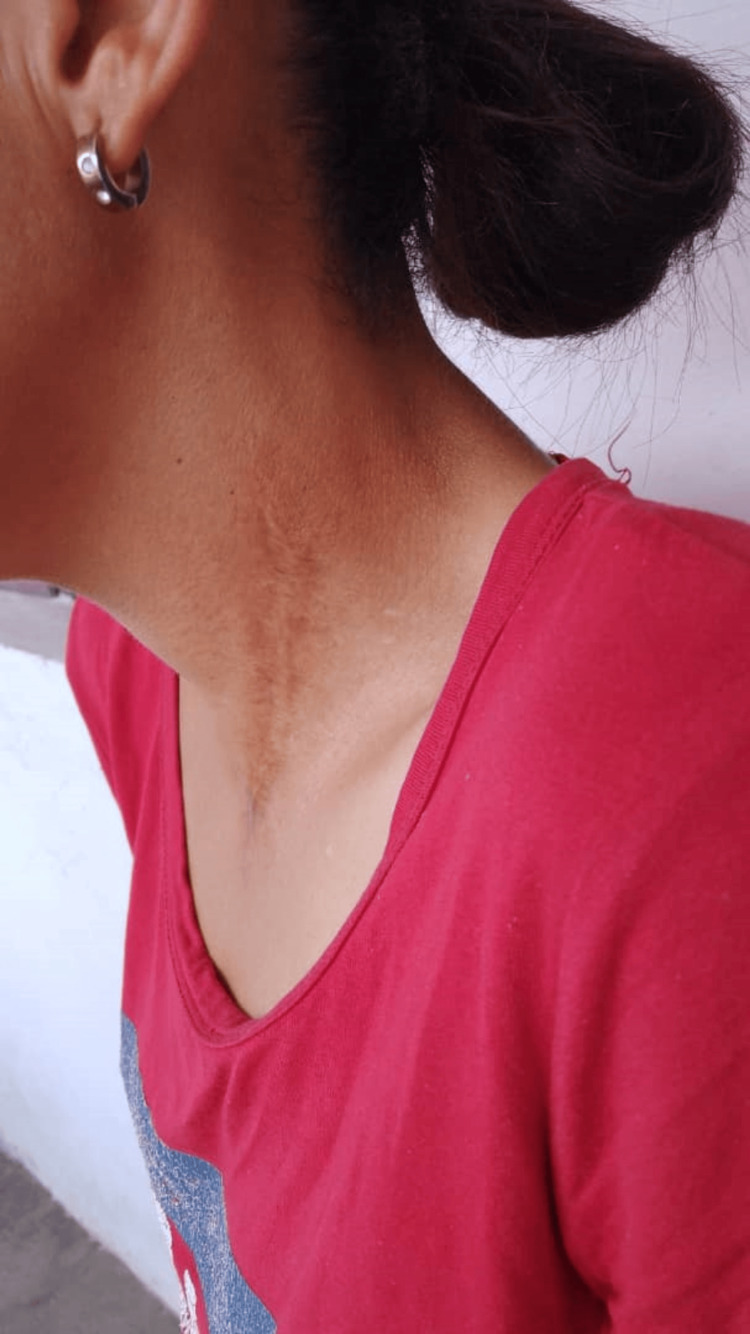
Case 3: Lateral views of postoperative scar after excision of the diverticulum.

The pathology report on the excised specimen described a segment of tissue consistent with the esophageal wall, including only mucosal and submucosal layers and areas of ulcerated squamous epithelium with muscle layers absent. The patient was maintained on treatment for gastroesophageal reflux. Sixteen years of postoperative follow-up showed no recurrence of the diverticulum (Figure 3).

Case 4 (2020)

A 35-day-old female twin presented with coughing, dyspnea, and cyanosis when feeding. The symptoms did not respond to the previously prescribed gastroesophageal reflux therapy. The infant had a history of esophageal atresia with distal esophageal fistula (type C) and had been operated on the third day after birth. The fistula was closed with end-to-end anastomosis. The baby was discharged from the neonatal intensive care unit with a diagnosis of gastroesophageal reflux disease.

**Figure 4:**
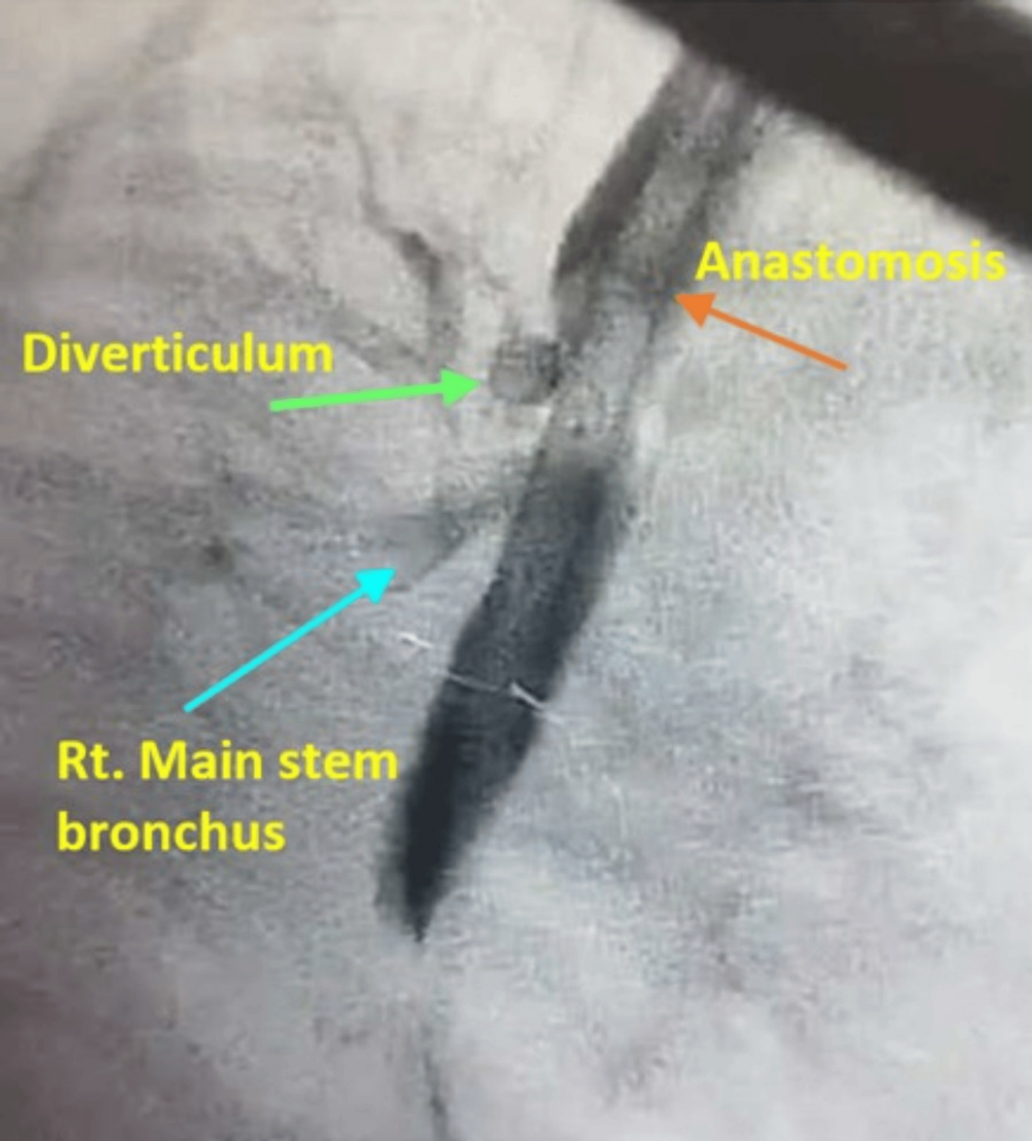
Case 4: Esophagogram showing an 8-mm rounded sac (green arrow), the site of anastomosis (red arrow), and the right main stem bronchus (blue arrow).

The presenting symptoms appeared 21 days after surgery. Upon examination at admission, signs of pneumonitis, predominantly in the right lung base, were found, raising suspicion of fistula recurrence. The esophagogram showed an 8-mm rounded sac protruding from the right anterolateral aspect of the esophageal wall above the stenotic lesion (marked before imaging) (Figure 4). There was leakage of contrast from the sac into the right main bronchus. The diagnosis of diverticulum was established based on these radiological findings.

Conservative management was not successful, and the respiratory condition deteriorated. During reoperation, a sac approximately 1 cm in diameter arose from the esophageal wall and adhered to the primary site of bronchial fistula insertion. Air leakage was detected when the sac was separated from the bronchial wall. The bronchial wall was closed. Due to the deteriorated state of the esophageal ends and poor respiratory status complicating anesthesia, esophageal re-anastomosis was not performed. The surgical team opted to perform a cervical esophagostomy and gastrostomy. The distal esophageal stump was closed. The patient died one month after the last surgery due to a brain hemorrhage secondary to congenital arteriovenous malformation (autopsy diagnosis).

## Discussion

An esophageal diverticulum (ED) is a protrusion of a part or the whole parietal structure of the esophageal wall outside the esophageal wall [1]. The diverticular pouch protrudes permanently at the weakest point of the esophageal wall, thus forming a pocket-like structure anywhere along the extension of the esophagus [14]. The diverticular pouch is usually filled with fluid and infected material.

ED is described according to the type (true or false), origin (congenital or iatrogenic), anatomical location (cervical, mid-esophageal, or epiphrenic), and etiopathology (pulsion or traction) [7]. A true ED comprises all esophageal wall layers, whereas a false diverticulum involves only the mucosa and submucosal layer [15]. Although congenital ED is usually presented in adulthood, there have been a few reported cases in children and premature infants [6]. Iatrogenic ED can occur following surgical repair of esophageal atresia and tracheoesophageal fistula [12]. Other causes of secondary ED in pediatrics include histoplasmosis [16] and foreign body impact [17]. ED may occur in the cervical region (hypopharyngeal ED), mid-esophagus, or lower region of the esophagus (epiphrenic diverticulum). Diffuse intramural pseudo-diverticulosis is the diffuse type involving the whole length of the esophagus and is associated with unfavorable prognoses [18]. Etiologically, ED can be due to causes that push the wall from inside, or pull it from outside, the lumen. Pulsion ED results from esophageal motility disorder with relatively increased pressure at an area of low parietal resistance [19]. Finally, traction ED is secondary to inflammation involving the mediastinum (peri-esophageal), resulting in adherence and eccentric traction on the esophageal wall [20]. Therefore, ED in pediatrics should be described in full detail for tailored management approaches.

This report presented a case of congenital diverticulum (Zenker’s diverticulum) at the age of 16 and five years. Additionally, two cases were presented with mid-esophageal ED as a complication of esophageal atresia and tracheoesophageal fistula repair. Most congenital ED is asymptomatic. Small-sized ED is usually presented in middle-aged adults and elder patients [8]. The most common symptom of ED is dysphagia, followed by regurgitation. Difficulty swallowing solid food and water characterizes cervical oropharyngeal dysphagia, e.g., Zenker’s diverticulum. Dysphagia in cervical ED is explained by inadequate relaxation of the cricopharyngeal muscle with subsequent narrowing of the upper esophageal sphincter.

Additionally, diverticular pouch compression exacerbates the narrowing of the sphincter [18]. Regurgitation occurs a few hours after a meal, depending on the level of the diverticulum. Other symptoms include respiratory symptoms such as chronic cough, stridor in infants, and respiratory distress due to chronic aspiration [2]. Furthermore, cervical ED can present with a cervical lump, with or without inflammation (diverticulitis) [3]. Left untreated, ZD can present a severe inflammatory condition, aspiration pneumonia, and broncho-esophageal fistula [13-14]. In infants and children, compromised nutritional status can significantly impact outcomes [15].

ZD is common among younger and older adults, with rare cases in children and infants [18]. This case series presents a young girl with an inflamed cervical mass, severe dysphagia, and regurgitation. The condition was complicated with septicemia and dehydration. Zenker’s diverticulum was diagnosed based on the presence of a cervical mass and an esophagogram. Additionally, histopathological assessment of the excised tissue confirmed the presence of mucosa and submucosal esophageal layers, thus confirming the diagnosis. This case series also included a five-year-old child who had caustic ingestion and esophagocoloplasty. The diverticulum was detected in the pharyngoesophageal remains. Contrary to the other cases, the symptoms were tolerable and managed conservatively. ZD is an acquired protrusion of the mucosa and submucosa of the pharyngoesophageal junction through the posterior wall known as Killian's triangle (also Killian’s dehiscence). Therefore, it is considered a pseudo-diverticulum [20]. ZD can be associated with other esophageal anomalies, including fistula and atresia. The mechanism of ZD protrusion is a matter of debate. However, the most implicated theory involves the abnormal functioning of the cricopharyngeal muscle and uncoordinated inferior pharyngeal constrictor muscle [18]. As a result, the esophageal intraluminal pressure increases leading to the herniation through the weakest point [20].

ED is an infrequent complication of esophageal atresia and tracheoesophageal fistula (EA/TEF). The reported incidence of ED following esophageal repair in EA/TEF cases varies. One study suggests a prevalence of 0.2% among 498 patients [13], while another reported 1.9% from 56 patients [14]. A recent study identified four out of 198 EA/TEF repair cases and performed thoracoscopic esophageal diverticulectomy [12]. Interestingly, in another study, none of the 92 cases following surgery for EA/TEF were reported to have ED complications [13].

The primary presenting symptoms of ED were dysphagia, cough, pneumonia, and malnutrition. In our case report, two instances involved ED complicating anastomosis surgery of EA/TEF. Two cases of ED were observed. The patient in case 2 had an ED near the anastomosis site, which was partially excised, and no further complaints were reported after five years of follow-up. ED developed postoperatively in case 4 and resulted in severe respiratory distress and pneumonia. The patient eventually succumbed to other causes despite successful surgery.

A barium swallow esophagogram is the gold standard radiographic tool to evaluate patients with suspected ED [5]. An esophagogram can detect the location and the size of the diverticulum. The delineated anatomy from the esophagogram provides the base for the subsequent operative intervention [19]. A recent study concurred that an esophagogram is fundamental in detecting ED [6]. Furthermore, broncho-esophageal fistula, usually associated with ED surgical complications, can be easily detected by an esophagogram [20]. For thoracic masses, computed tomography (CT) can be used to detect the location and measure the size of thoracic masses, revealing hypodense lesions consistent with cysts. Magnetic resonance imaging (MRI) and micro-laryngo-bronchoscopy (MLB) can assess lesions and mediastinal anomalies. In case of suspected upper airway obstruction, flexible bronchoscopy is a valuable tool to detect retropharyngeal masses, obstruction sites, and fistulae opening [7]. For cases of stridor in newborns and infants, MLB helps detect the cause, including cysts. An esophagogram was this study's primary diagnostic tool for all four reported cases. The location, size, and type of fistula guided the intervention.

Surgical intervention plays a pivotal role in managing patients with symptomatic ED. However, there is no consensus on managing mild and asymptomatic patients. Therefore, careful follow-up is mandatory for high-risk patients and those with a large diverticulum [20]. Myotomy is considered the primary surgical procedure for pediatric ED management. Numerous studies report excellent success rates and reduced recurrence [20]. Typically, cricopharyngeal myotomy is performed via the left anterior cervical approach [7]. Evaluation of the surrounding structures for possible fistula formation is crucial [6,19]. Diverticulopexy is considered in select cases [18]. Minimally invasive surgery utilizing laparoscopy or thoracoscopy has also been progressively developed for ED management [20].

Intraluminal esophagoscopy represents a promising approach to managing ED. The technique disrupts the connection between the diverticulum and the esophageal lumen [20]. The procedure involves coagulation followed by division and stapling resection of the common wall [7]. However, its application in pediatric cases requires further investigation.

Although the endoscopic approach is safe and effective, it is not suitable for large diverticula because these are more likely to result in incomplete pouch emptying [6]. One case (case 1) had a small diverticulum and wide ostium, requiring conservative management. Remarkably, this patient had an uneventful clinical course during the 20-year follow-up. In other cases involving a large inflamed cervical supraclavicular mass, drainage of the diverticulum was performed. Diverticulectomy and myotomy were the main surgical interventions in these cases. During surgery, the integrity of the bronchial wall was evaluated, and the identified fistula was closed. In one instance, the diverticulum is partly excised, and the distal segment is utilized to repair the esophagus to rectify potential lumen stenosis distal to the diverticulum.

Physicians and surgeons should pay attention to pediatric ED care. It should never be overlooked among the differential diagnoses. A deeper understanding of ED's significance to the overall health of infants and children enables earlier diagnosis and care of more complicated cases of ED, hence reducing the risk of unfavorable outcomes.

## Conclusions

Esophageal diverticula are rare in infants and children. These are typically congenital or iatrogenic, which complicates EA/TEF. Congenital ED is thought to result from upper-esophageal sphincter pressure abnormality. Clinical presentations include dysphagia, regurgitation, and respiratory distress. Stridor is a common feature of ZD in newborns. Inflammation and sepsis complicate large ZD presented as a supraclavicular mass. Although a barium esophagogram is the gold standard diagnostic procedure, other modalities, including ultrasound, CT scan, MRI, or histopathology, can confirm the diagnosis. The mainstay of surgical interventions is diverticulectomy, but cervical diverticula are treated by cricopharyngeal myotomy. Conservative management can be considered in select cases. ED is extremely rare. Nonetheless, it should always be considered in the differential diagnosis of patients presenting pharyngoesophageal and tracheal symptoms.
